# Novel endolysin LysMP for control of *Limosilactobacillus fermentum* contamination in small-scale corn mash fermentation

**DOI:** 10.1186/s13068-023-02400-5

**Published:** 2023-09-29

**Authors:** Maulik H. Patel, Shao-Yeh Lu, Siqing Liu, Christopher D. Skory

**Affiliations:** 1 Oak Ridge Institute for Science and Education (ORISE), Oak Ridge, TN USA; 2 USDA, Agricultural Research Service, National Center for Agricultural Utilization Research, Renewable Product Technology Research Unit, 1815 N. University St, Peoria, IL 61604-3902 USA

**Keywords:** Endolysin, Peptidoglycan hydrolase, Bioethanol, *Lactobacillus*, Antimicrobial, Biorefineries, Corn mash fermentation, *Limosilactobacillus*

## Abstract

**Background:**

Traditional bioethanol fermentation industries are not operated under strict sterile conditions and are prone to microbial contamination. Lactic acid bacteria (LAB) are often pervasive in fermentation tanks, competing for nutrients and producing inhibitory acids that have a negative impact on ethanol-producing yeast, resulting in decreased yields and stuck fermentations. Antibiotics are frequently used to combat contamination, but antibiotic stewardship has resulted in a shift to alternative antimicrobials.

**Results:**

We demonstrate that endolysin LysMP, a bacteriophage-encoded peptidoglycan hydrolase, is an effective method for controlling growth of LAB. The LysMP gene was synthesized based on the prophage sequence in the genome of *Limosilactobacillus fermentum* KGL7. Analysis of the recombinant enzyme expressed in *E. coli* and purified by immobilized metal chelate affinity chromatography (IMAC) showed an optimal lysis activity against various LAB species at pH 6, with stability from pH 4 to 8 and from 20 to 40 °C up to 48 h. Moreover, it retains more than 80% of its activity at 10% ethanol (v/v) for up to 48 h. When LysMP was added at 250 µg/mL to yeast corn mash fermentations containing *L. fermentum*, it reduced bacterial load by at least 4-log fold compared to the untreated controls and prevented stuck fermentation. In comparison, untreated controls with contamination increased from an initial bacterial load of 1.50 × 10^7^ CFU/mL to 2.25 × 10^9^ CFU/mL and 1.89 × 10^9^ CFU/mL after 24 h and 48 h, respectively. Glucose in the treated samples was fully utilized, while untreated controls with contamination had more than 4% (w/v) remaining at 48 h. Furthermore, there was at least a fivefold reduction in lactic acid (0.085 M untreated contamination controls compared to 0.016 M treated), and a fourfold reduction in acetic acid (0.027 M untreated contamination controls vs. 0.007 M treated), when LysMP was used to treat contaminated corn mash fermentations. Most importantly, final ethanol yields increased from 6.3% (w/v) in untreated contamination samples to 9.3% (w/v) in treated contamination samples, an approximate 50% increase to levels comparable to uncontaminated controls 9.3% (w/v).

**Conclusion:**

LysMP could be a good alternative to replace antibiotics for mitigation of LAB contamination in biofuel refineries.

**Supplementary Information:**

The online version contains supplementary material available at 10.1186/s13068-023-02400-5.

## Background

The biofuel industry has experienced steady growth over the past few decades due to the increasing cost of petroleum and renewable fuel standards. By August 2022, the bioethanol production capacity in the United States has surpassed 15.6 billion gallons and is projected to reach 60 billion gallons per year by 2030 to meet the increasing need for ethanol blending in gasoline [1, 2]. Bacterial contamination remains a major concern at bioethanol fermentation facilities, as chronic and acute contamination occurs frequently, leading to stuck fermentation [4, 5]. These events often necessitate the shutdown of facilities for cleaning and result in significant economic loss due to contamination [6, 7]. Microbial analysis of these facilities has shown that lactic acid bacteria (LAB) are the most prevalent contaminant [5, 6]. More specifically, *Limosilactobacillus fermentum* and phylogenetically related heterofermentative LAB species have been identified as major culprits causing stuck fermentation across bioethanol facilities [6, 8]. To minimize contamination at the facilities, hop acids, chlorine gas and antibiotics are often used prophylactically to treat active contamination [9–11]. Antibiotic residues in distillery products, such as distillers dried grains have been reported, raising concerns on potential antibiotic resistance and contamination in animal feed [12, 13]

Endolysins are lytic enzymes of bacteriophage that, with the help of holins (pore forming transmembrane protein), lyse infected host cells and release viral particles during a lytic infection cycle [14]. Endolysins have the potential to be an excellent alternative to antibiotics because bacteria theoretically are unable to develop resistance against endolysin and they target a narrow group of microbes [15, 16]. Endolysins against Gram-positive bacteria typically contain cell wall enzyme activity domain (EAD) for cell wall lysis and cell wall binding domain (CBD) while, endolysins against Gram-negative bacteria generally lack the CBD domain [17, 18]. In this work, we searched for potential endolysin genes using an endolysin specific domain associated with prophage sequences found in *L. fermentum* KGL7 (isolated from rice beverages of Northeast Indian tribe (SRA accession number: PRJNA563537) [19]. LysMP with a putative GH25 enzymatically active domain and a SH3b homologue cell wall binding domain was identified, synthesized and recombinantly expressed in *Escherichia coli*. The purified endolysin was characterized for lytic activity against various LAB strains, optimum activity in various conditions and its potential to be used as an antimicrobial agent in biorefineries to mitigate LAB contamination.

## Results

### Identification of LysMP gene and lytic activity of purified endolysin

**Fig. 1:**
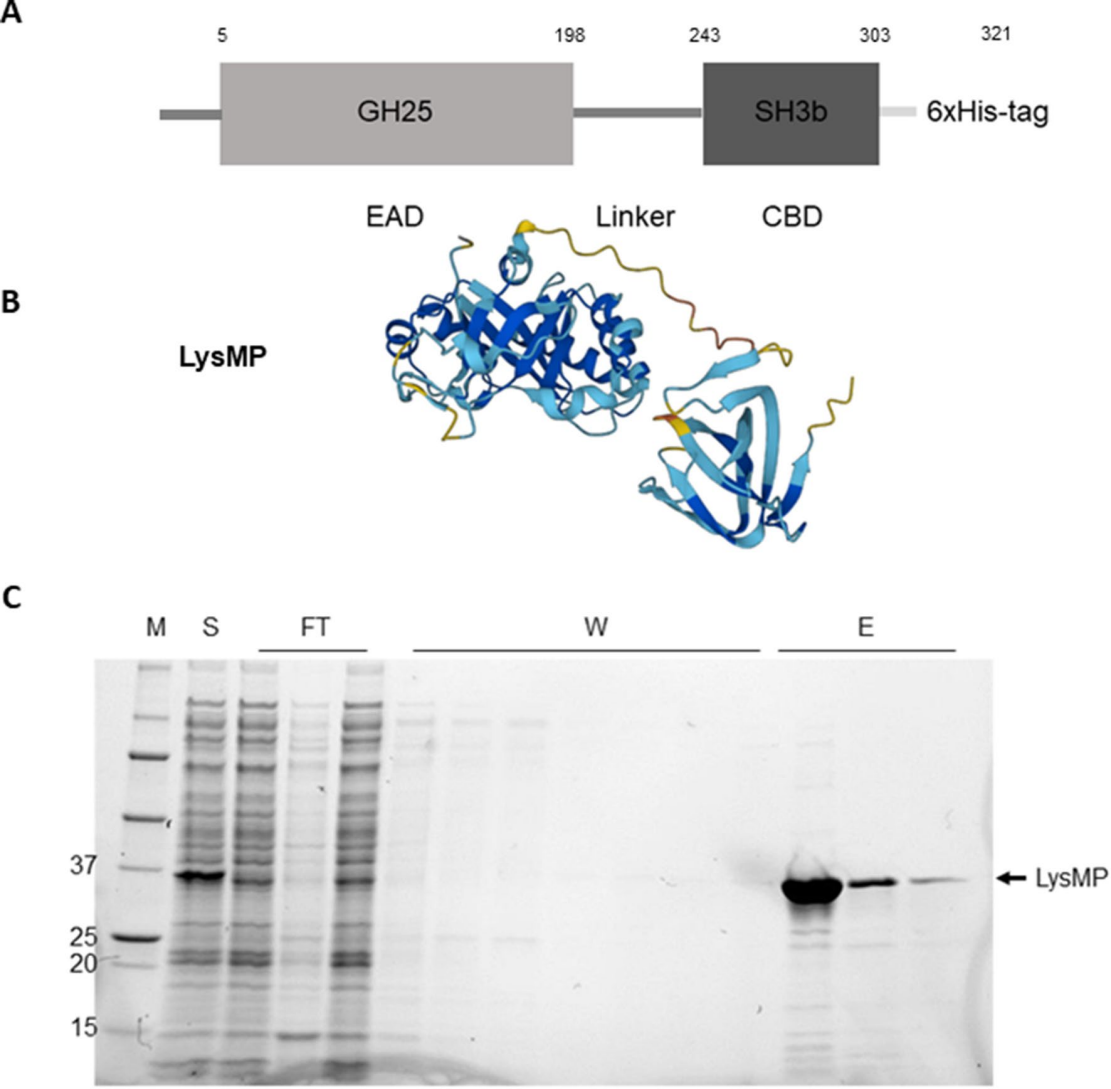
Schematic representation of LysMP domain structures. **A** The LysMP conserved domain representation depicting endolysin active domain (EAD) and cell wall binding domain (CBD) joined by a linker. **B** Three-dimensional model structure predicted by ESMFold [22]. **C** The SDS-PAGE gel of the purified recombinant LysMP. Marker (M; Precision Plus Protein Standard; Bio-Rad), whole cell lysate of induced LysMP (S), Flow through (FT) during IMAC purification, wash (W), and Elution (E) is the eluted protein after IMAC purification of carboxyl-terminal 6 × histidine-tagged LysMP (35.9 kDa)

The endolysin LysMP gene (GenBank accession number OQ298930) was identified from the genomic sequence of *L. fermentum* KGL7, previously isolated from rice beverage, by Pfam domains specific to phage-associated glycoside hydrolase family 25 (GH25) [20, 21]. The gene was codon optimized for *E. coli* expression, synthesized, and cloned into expression vector, pET-21a (+) (GenScript). The LysMP consists of an active catalytic muramidase-superfamily domain (GH25, EAD) and a cell wall binding SH3b homologue domain (CBD; Fig. 1A). Predicted LysMP structure obtained utilizing ESMFold [22] demonstrated the typical EAD-linker-CBD structure (Fig. 1B). Based on the domain functionality, LysMP likely cleaves β-(1,4)-glycosidic bond of the peptidoglycan N-acetylglucosamine–N-acetylmuramic acid (NAG–NAM) linkages. We were able to express and purify the carboxyl-terminal 6xHis-tagged recombinant lytic protein in *E. coli* using a fast protein liquid chromatography (FPLC; Äkta pure^™^, Cytiva) and confirm a predicted molecular mass of 35 kDa (321 aa; Fig. 1C) by SDS-PAGE analysis.

### LysMP concentration-dependent lytic activity

**Fig. 2:**
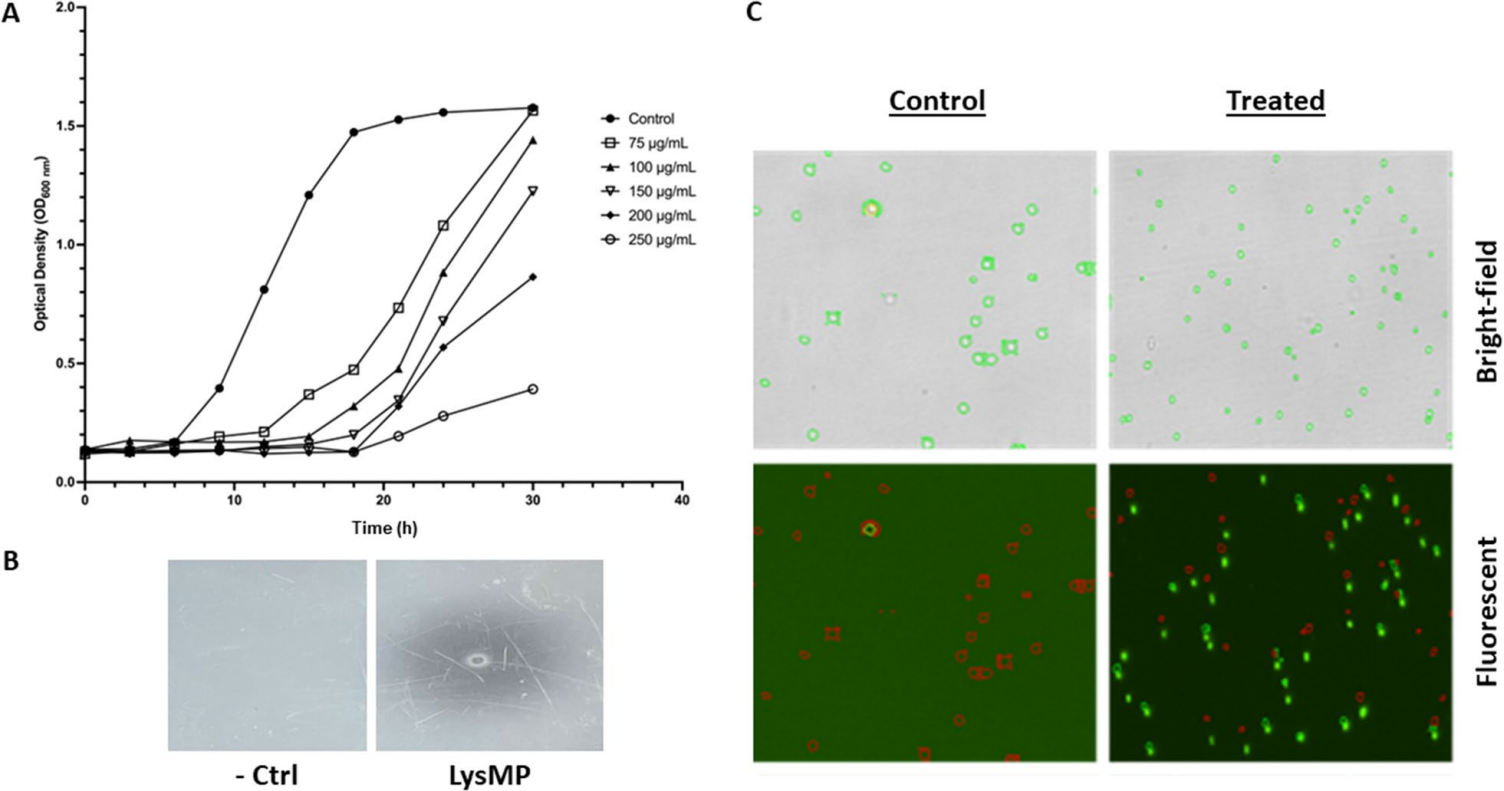
Endolysin LysMP inhibition of susceptible bacteria. **A** Dose-dependent growth inhibition of purified LysMP against *L. fermentum* 0315-25 over time. **B** Antimicrobial spot assay of LysMP on polyacrylamide gel with target bacterium. Sterile 1 × PBS served as negative control (-Ctrl) and purified endolysin LysMP as treatment (LysMP). **C** Bacterial viability assay. Target bacterium with and without 30 min LysMP treatment (100 µg/mL). Bright-field images detect all cells. Green, fluorescent signal detect dead bacteria with compromised cell membrane. Red cells detect live bacterial cells

Growth delay was observed when different concentrations of purified LysMP (75, 100, 150, 200 and 250 µg/mL) were used to treat the target bacterium, *L. fermentum* 0315-25 (Fig. 2A**)**. When compared to no treatment control at the end of 24 h (OD_600_ = 1.6), the growth of bacteria was significantly suppressed (OD_600_ = 0.4) and delayed at 250 µg/mL concentration (Fig. 2A). The lytic activity of LysMP was validated with spot plate assay by aliquoting 5 µL (1 µg/µL) of purified endolysin on polyacrylamide gel polymerized with *L. fermentum* 0315-25. The visual observation of the zone of clearing with the purified LysMP validated the lytic potential of the endolysin when compared to the PBS negative control (Fig. 2B). The endolysin’s lytic activity was further confirmed with bacterial viability assay using SYTOX^®^ dye and counting cells with a Cellometer cell counter. SYTOX^®^ nucleic acid stain penetrates compromised bacterial cell membranes but does not cross intact cell membranes of live cells. The treatment control showed very few dead cells (< 3%), while more than 90% of cells stained positive with SYTOX, after 30 min of LysMP (100 µg/mL) treatment (Fig. 2C), which further confirmed the lytic activity of LysMP against target bacteria.

### Biochemical characterization of LysMP

**Fig. 3:**
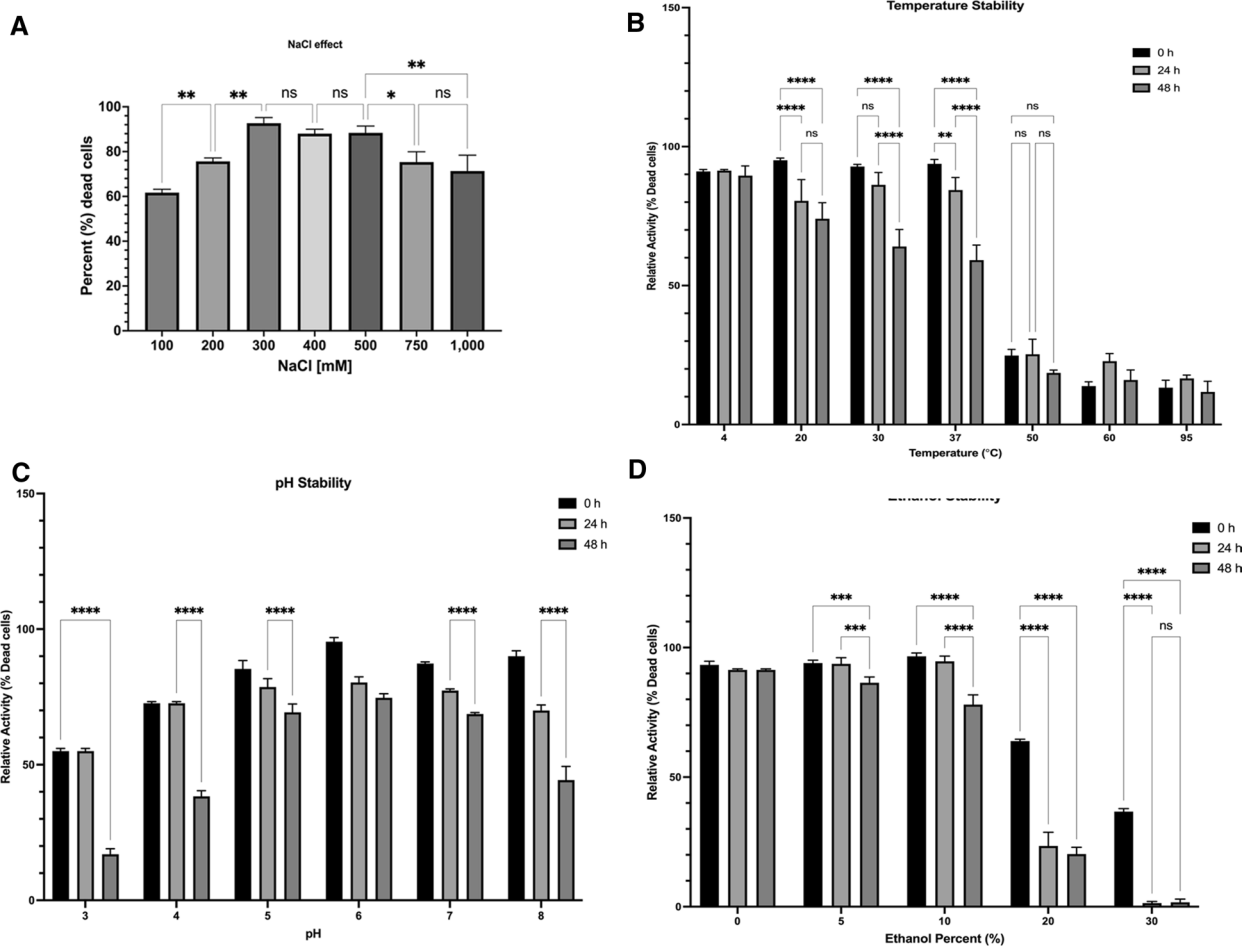
LysMP stability at different salt concentration, temperature, ethanol, and pH. **A** Effect of sodium chloride, NaCl concentrations [0–1000 mM] on the enzymatic activity of endolysin LysMP against *L. fermentum* 0315-25. The enzymatic activity was determined using growth inhibition assay where percent (%) dead cells were determined by Cellometer measurement using SYTOX stain. The LysMP stability was evaluated by subjecting LysMP (100 µg/ml) to various conditions for a period of 0, 24, and 48 h, followed by activity measurements using viability assays against *L. fermentum* 0315-25. Relative activity is expressed as a normalized percent dead cell. Data are means ± standard deviations (*n* = 3) using analysis of variant. *p < 0.05; **p < 0.01; ****p < 0.00001. **B** LysMP subjected to 0 ℃, 20 ℃, 30 ℃, 37 ℃, 50 ℃, 60 ℃ and 95 ℃. **C** LysMP exposed to pH 3, 4, 5, 6, 7 and 8. **D** LysMP exposed to 0–30% (v/v) ethanol concentration. All data are means ± standard deviations with three independent biological replicates (*n* = 3) and statistically analysed using analysis of variant where not statistically significant (ns); **p < 0.01; ****p < 0.00001

The ionic strength of the buffer is one of the key parameters to optimize the activity of endolysin [23]. The LysMP activity was examined in the presence of 0.1–1 M salt concentration (Fig. 3A**)**. The optimal range of sodium concentration was determined to be from 0.3 to 0.5 M, with the biggest difference being between 0.1 and 0.5 M NaCl (*p* < 0.01). Further increase of NaCl concentration from 0.5 to 0.7 5 M (*p* < 0.05) and 1 M (*p* < 0.01) resulted in a significant decrease in LysMP lytic active. No significant differences in the enzymatic activity were observed between 0.5 and 1 M NaCl.

### LysMP endolysin stability under fermentation environmental stress

We examined the stability profile of LysMP by subjecting the purified enzyme under stress conditions commonly found in industrial bioethanol fermentation facilities. The enzymatic activity was analysed by bacterial viability assay as described in the method section following treatment under a range of temperature, pH, and ethanol concentrations for 0, 24, and 48 h. Thermostability of the enzyme was observed from 4 to 95 °C for at least 48 h (Fig. 3B). LysMP was stable in the range of 20–37℃ for at least 24 h. Significant decrease in enzymatic lytic activity can be observed at 48 h when comparing to 0 h at 20 ℃, 30 ℃, and 37 ℃ (*p* < 0.0001). There were no significant differences in LysMP activity when comparing between different time points (0, 24 and 48 h) at 50 °C. Significant decrease in lytic activity was observed at around 20% and continued to deteriorate as temperatures were increased to 60 ℃ and 95 ℃ for up to 48 h. Under various pH conditions, LysMP performed optimally at pH 6 where no significant loss in lytic activity was observed up to 48 h (Fig. 3C). The enzyme was functionally stable in a range of pH 4–8, up to 24 h. The relative lytic activity was significantly reduced to about 55% at pH 4. In all pH conditions tested at the 48-h time point, LysMP’s lytic activity were significantly impacted when comparing to time 0 h and 24 h (*p* < 0.0001) except for pH 6. When purified LysMP is exposed to ethanol, it is at least 80% stable in 10% (v/v) ethanol concentration for up to 48 h. (Fig. 3D). The lytic activity significantly decreased starting at 20%, and at 30% (v/v) ethanol concentration. Values dropped to less than 40% within the first hour (0 h) and substantially decreased to below 10% activity for 24 and 48 h when compared to 0-h time point (*p* < 0.0001).

### Divalent cation binding prediction of LysMP

**Fig. 4:**
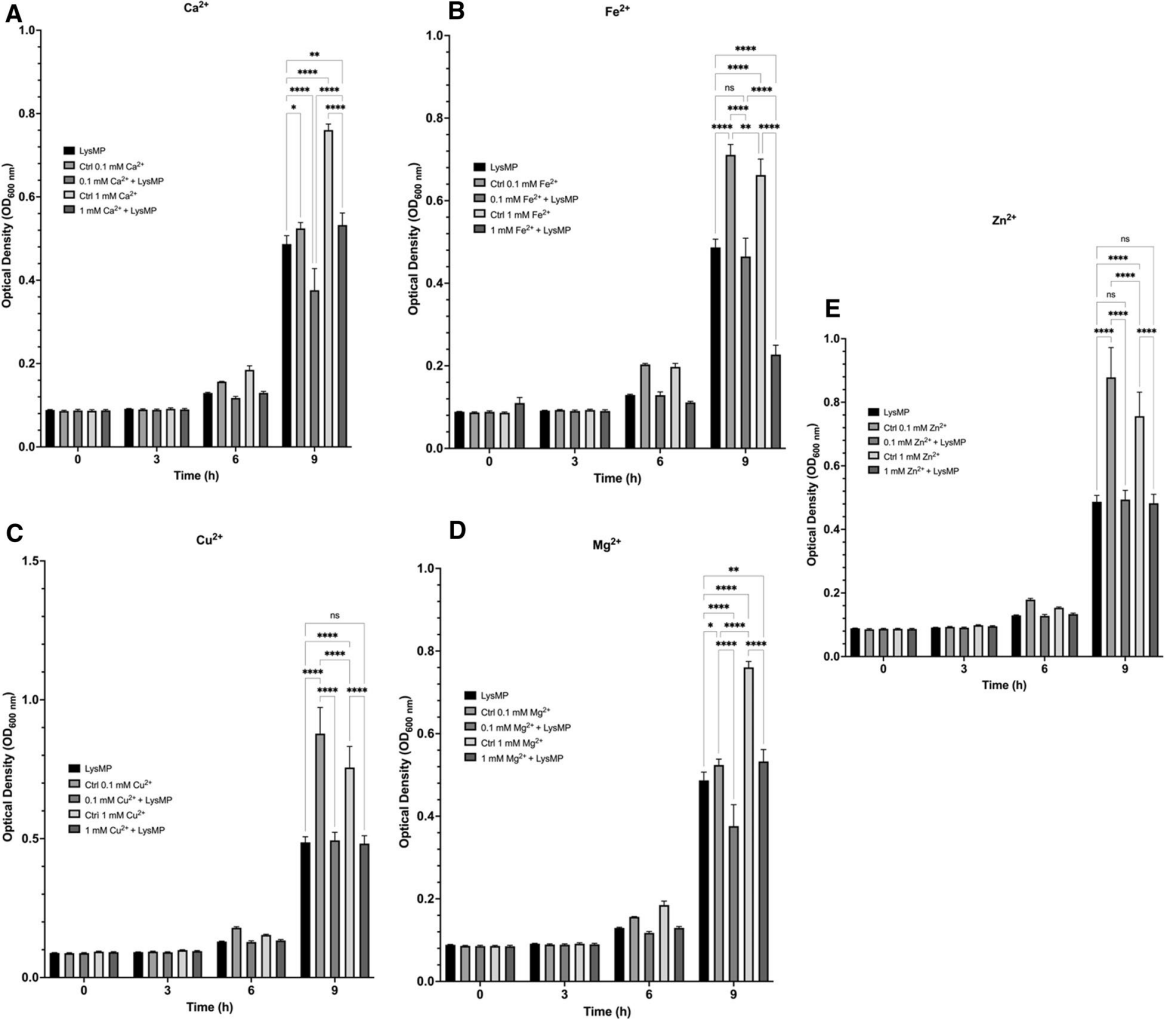
Biochemical characterization of LysMP. **A** Calcium ion (CaCl_2_) at 0.1 mM and 1 mM were added to endolysin LysMP treatment. **B** Copper ion (CuCl_2_) at 0.1 mM and 1 mM were added to endolysin LysMP treatment and OD_600_ was used to determined growth of target bacteria over a period of 9 h at 37℃. **C** Iron(II) (FeCl_2_) at 0.1 mM and 1 mM were added to endolysin LysMP treatment. **D** Magnesium iron (MgCl_2_) at 0.1 mM and 1 mM were added to endolysin LysMP treatment. **E** Zinc iron (ZnCl_2_) at 0.1 mM and 1 mM were added to endolysin LysMP treatment. The OD_600_ was used to determined growth of target bacteria *L. fermentum* 0315-25 over a period of 9 h at 37 ℃. Data are means ± standard deviations (*n* = 3) using analysis of variant. *p < 0.05; **p < 0.01; ****p < 0.00001

The LysMP activity in the presence of divalent cations were examined (Fig. 4). The growth of *L. fermentum* was measured for 9 h at 37 °C in the presence of divalent cations Ca^2+^, Cu^2+^, Fe^2+^, Mg^2+^, and Zn^2+^ at 0.1 mM and 1 mM concentration, with and without the presence of LysMP (Fig. 4A–E**)**. Significantly more growth was observed with the addition of Ca^2+^ at 0.1 mM (*p* < 0.05) and 1 mM (*p* < 0.0001) compared to LysMP alone (Fig. 4A). When both LysMP and Ca^2+^ were added as treatment, a significant reduction of *L. fermentum* was observed at 0.1 mM (*p* < 0.0001), but not at 1 mM concentration. Similar trends were observed when comparing endolysin only treatment to addition of Cu^2+^ at 0.1 mM and 1 mM (*p* < 0.0001) and its cation only controls (0.1 mM and 1 mM without endolysin); Fig. 4B). LysMP showed optimum activity in the presence of 1 mM Fe^2+^ when compared with LysMP control only (*p* < 0.0001; Fig. 4C). While the improved lytic activity was observed in the presence of 0.1 mM Mg^2+^ (*p* < 0.0001; Fig. 4D), no significant effect was detected with addition of Zn^2+^ at any concentration (Fig. 4E).

### The LysMP lytic potential against lactic acid bacteria

**Fig. 5:**
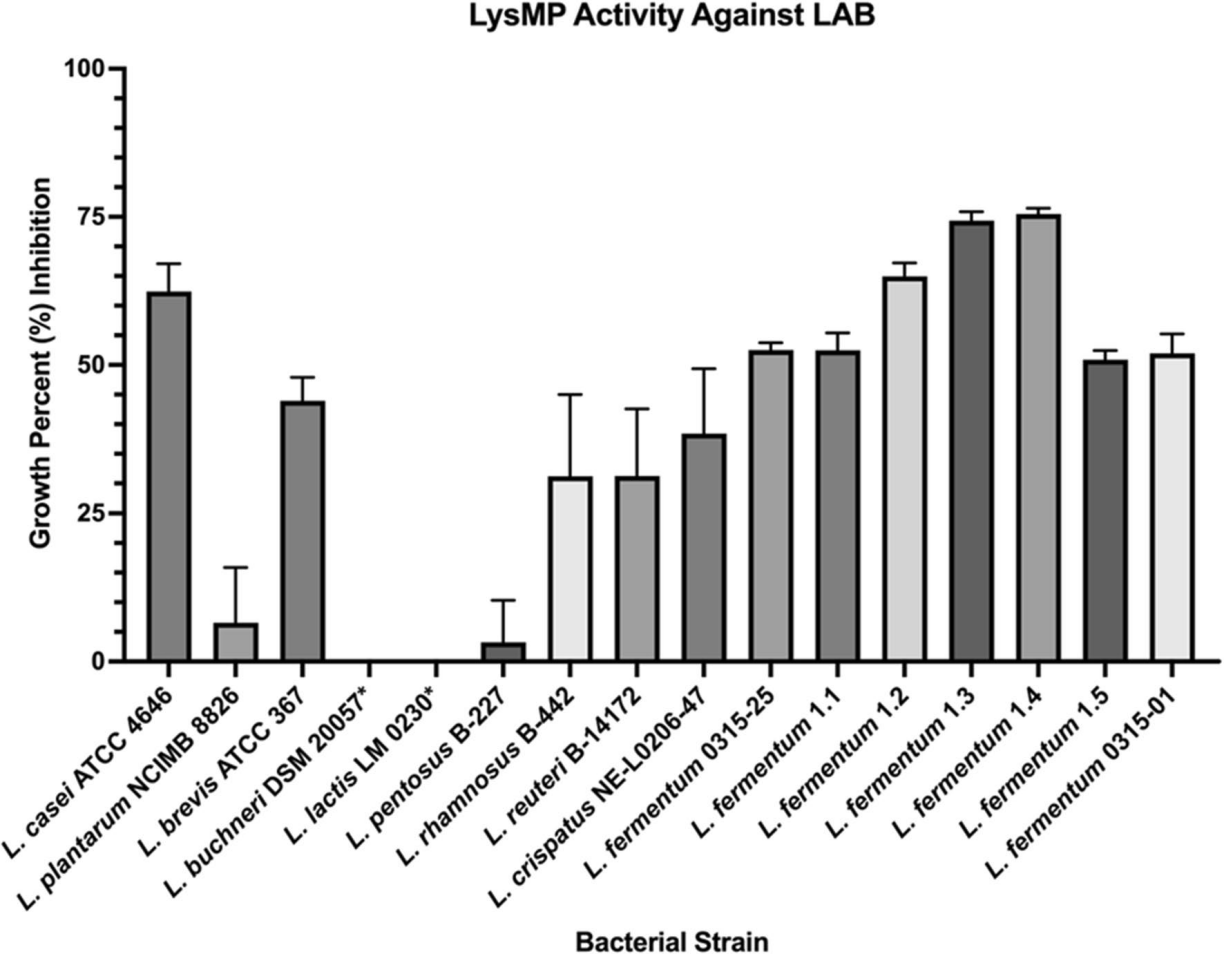
LysMP activity against various LAB strains. LysMP at 100 µg/mL was tested for activity against the various lactic acid producing species and bioethanol isolates. Activity of LysMP was measured by percent (%) growth inhibition of susceptible bacteria strains. Asterisk (*) strains are below detectable limit. Data are means ± standard deviations with three independent biological replicates (*n* = 3)

Growth inhibition assay for the LysMP was conducted to test inhibition potential against several reference LAB species and isolates from commercial fuel ethanol fermentation plant (Fig. 5**; **Table 1). Exogenous addition of the purified endolysin at 100 µg/mL resulted in more than 50% growth reduction in all tested *L.* f*ermentum* strains grown in the MRS media after 24 h at 37℃. In addition, purified LysMP showed greater than 60% growth inhibition against *Lacticaseibacillus casei* ATCC 4646 strain while 25% to 45% growth inhibition were observed against *Lactobacillus brevis* ATCC 367 (44%) *Lactobacillus rhamnosus* B-442 (32%), *Lactobacillus reuteri* B-14172 (31%) and *Lactobacillus crispatus* NE-L0206-47 (39%). In contrast, less than 10% inhibition was observed for *Lactiplantibacillus plantarum* NCIMB 8826 (6%) and *Lactiplantibacillus pentosus* B-227 (3%). LysMP was completely ineffective against *Lentilactobacillus buchneri* DSM 20057 and *Lactococcus lactis* LM 0230 (Fig. 5).

**Table 1:** Bacterial and yeast strains used in this study

Strain	Relevant genotype/phenotype^a, b^	Reference or source^c^
*Escherichia coli*		
BL21(DE3)pLysS	F^−^ *ompT hsdS*_B_ (r_B_-m_B_-) *gal dcm* (DE3) pLysS (Cam^R^)	ThermoFisher
BL21(DE3)/pLysS/pET-21a(+)::LysMP	Amp^R^, Cam^R^, containing LysMP gene	GenScript, this study
E. cloni 10G/pRham N-His Kan::LysKB317	Kan^R^, containing LysKB317 gene	[1]
*Lacticaseibacillus casei*		
*L. casei* ATCC4646	Species reference strain	ATCC
*Lactiplantibacillus plantarum*		
*L. plantarum* NCIMB 8826	Species reference strain	NCIMB
*Lactobacillus brevis*		
*L. brevis* ATCC 367	Species reference strain	ATCC
*Lentilactobacillus buchneri*		
*L. buchneri* DSM 20057	Species reference strain	DSMZ
*Lactococcus lactis*		
*L. lactis* LM 0230	Species reference strain	NRRL, [55]
*Lactiplantibacillus pentosus*		
* L. pentosus* B-227	Biofuel contaminant wildtype^b^	NRRL
*Lacticaseibacillus rhamnosus*		
* L. rhamnosus* B-442	Biofuel contaminant wildtype^b^	NRRL
*Limosilactobacillus reuteri*		
* L. reuteri* B-14172	Biofuel contaminant wildtype^b^	NRRL
*Lactobacillus crispatus*		
* L. crispatus* NE-L0206-47	Biofuel contaminant wildtype^b^	NRRL
*Limosilactobacillus fermentum*		
* L. fermentum* 1.1	Biofuel contaminant wildtype^b^	This study [32]
* L. fermentum* 1.2	Biofuel contaminant wildtype^b^	This study [32]
* L. fermentum* 1.3	Biofuel contaminant wildtype^b^	This study [32]
* L. fermentum* 1.4	Biofuel contaminant wildtype^b^	This study [32]
* L. fermentum* 1.5	Biofuel contaminant wildtype^b^	This study [32]
* L. fermentum* 0315-01	Biofuel contaminant wildtype^b^	[1]
* L. fermentum* 0315-25	Biofuel contaminant wildtype^b^	[1]
*Saccharomyces cerevisiae*		
* S. cerevisiae* NRRL Y-2034	Bioethanol producing strain	NRRL, [56]

^a^*Amp*^*R*^ ampicillin-resistant; *Cam*^*R*^, chloramphenicol-resistant; *Kan*^*R*^, kanamycin-resistant

^b^Biofuel contaminant wildtype were isolated from a Midwestern dry-grind fuel ethanol facility from a previous screen [32, 57]

^c^USDA-ARS Culture Collection (NRRL); German collection of microorganisms and cell cultures (DSMZ); National collection of industrial food and marine bacteria (NCIMB)

### Endolysin LysMP reduced *L. fermentum* in corn mash fermentation

**Fig. 6:**
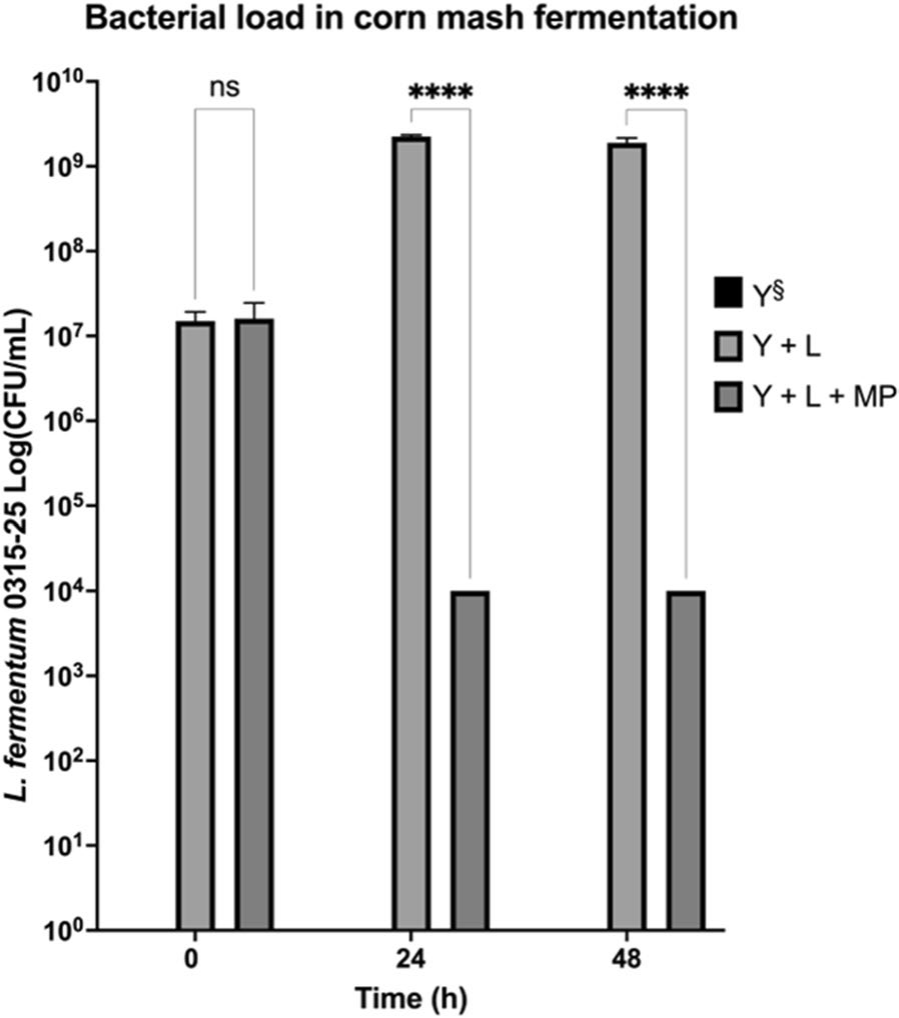
Bacterial count in model corn mash fermentation. The *L. fermentum* bacterial count was measured in the corn mash samples by plating on MRS medium supplemented with cycloheximide as yeast inhibitor. Y: yeast control (no contamination; black bar), Y + LF: *L. fermentum* inoculated along with yeast (contamination challenge; light grey bar), Y + LF + LMP: LysMP endolysin was supplemented into bacterial infected corn mash and yeast (dark grey bar). Section sign (§) indicates yeast control (black bar) had no measurable presence of *L. fermentum*. All data are means ± standard deviations with two independent biological replicates (*n* = 2) and statistically analysed using analysis of variant where not statistically significant (ns); ****p < 0.00001

We evaluated the efficiency of LysMP to reduce *L. fermentum* 0315-25 contamination using a small-scale corn mash fermentation model (Fig. 6). In bacterial contaminated fermentation flasks (Y + L + MP), more than 4-log reduction of bacterial load was observed (below detection limit of 5-log CFU/mL) when purified LysMP at 250 µg/mL was exogenously added compared to the no treatment infection control (Y + L) at 24 h and 48 h (*p* < 0.0001). In no treatment flasks, the bacterial count increased to 2.25 × 10^9^ CFU/mL from the initial 1.50 × 10^7^ CFU/mL throughout the 48-h fermentation period. In yeast only control (Y) fermentation, no bacteria were detected.

### Corn mash fermentation analysis

**Fig. 7:**
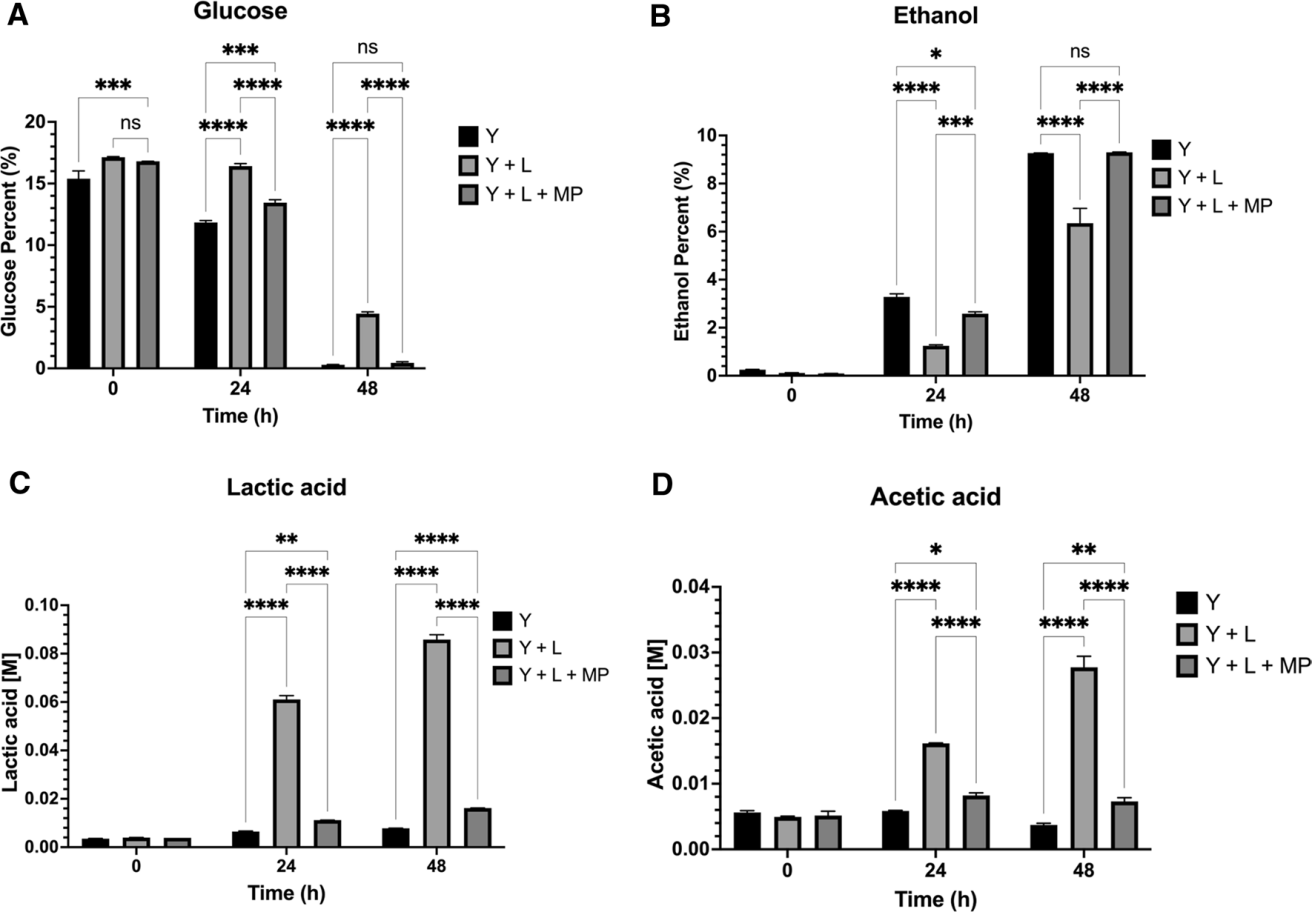
Corn mash sugar consumption and analyses of fermentation products. Corn mash fermentation analysis of **A** glucose, **B** ethanol, **C** lactic acid, and **D** acetic acid for: yeast control (Y; no bacterial infection), *L. fermentum* infection with yeast (Y + LF; contamination challenge), and LysMP endolysin supplemented yeast with bacterial infection (Y + LF + LMP). All data are means ± standard deviations with two independent biological replicates (*n* = 2) and statistically analysed using two-way ANOVA. *p < 0.05; ***p < 0.0001; ****p < 0.00001 and ns = not statistically significant

Glucose utilization, ethanol production, lactic acid, and acetic acid were analysed at each time point (0, 24, and 48 h; Fig. 7A–C). Percent glucose in yeast (Y) and the treatment (Y + L + MP) showed no significant differences in utilization at the end of 48 h fermentation (> 0.4% (w/v); however, when compared to the infection control (Y + L) at 48 h, a significant difference was observed (*p* < 0.0001) leaving less than 5% (w/v) glucose unused (Fig. 7A). Percent ethanol generated at the end of 48 h, demonstrated yeast control and treatment (Y + L + MP) had no significant difference (9.3% (w/v); Fig. 7B). Only 6. 3% (w/v) ethanol was generated by the infected flask (Y + L; *p* < 0.0001). At 48 h, both lactic acid (0.008–0.016 M) and acetic acid (0.004–0.008 M) concentration in yeast and LysMP treatment (Y + L + MP) showed significantly lower levels (*p* < 0.0001) than bacterial infection samples for lactic acid (0.085 M) and acetic acid (0.028 M; Fig. 7C, D).

### Synergistic effect of combined endolysin in infected corn mash

**Fig. 8:**
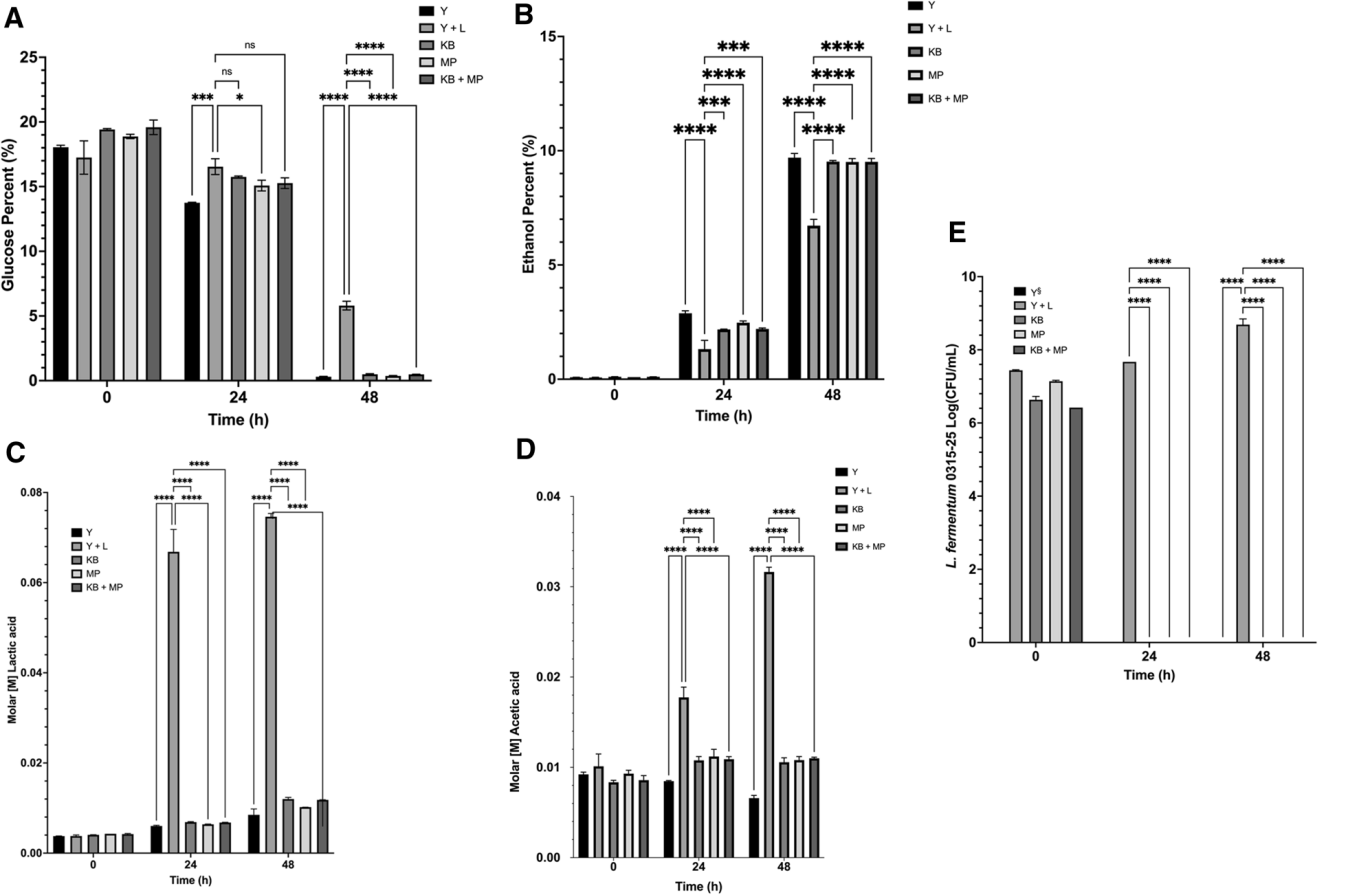
Analysis of fermentation products with crude cell lysate LysMP and LysKB317 treated corn mash fermentation. **A** Percent glucose (%), **B** percent (%) ethanol, **C** molar [M] concentration of lactic acid, **D** molar [M] concentration of acetic acid, **E** log CFU/mL of *L. fermentum* 0315-25 in corn mash fermentation. Section sign (§) indicates yeast control (black bar) has no measurable presence of *L. fermentum*. Treatment of crude lysate of estimated 250 µg/mL endolysin LysMP and/or LysKB317 were added into 20 mL corn mash fermentation (*n* = 2 independent replicates and error bar = SD)

The introduction of crude lysate LysMP (~ 250 µg/mL; MP) and crude lysate LysKB317 (~ 250 µg/mL; KB), both independently and in combination (~ 250 µg/mL; MP + KB), resulted in significant difference (*p* < 0.0001) in glucose utilization, ethanol generation, lactic acid and acetic acid concentrations compared to infection control (Y + L); Fig. 8A**–**D. Bacterial load were below the detection limit in yeast only control (Y) and crude lysate treatment (MP, KB, MP + KB; Fig. 8E). A significant difference (*p* < 0.0001) in bacterial load for infection control (Y + L) was observed (8.7 log CFU/mL) compared to endolysin treatment and control at the end of 48 h (below detection). No synergistic nor additive effect was observed between the addition of crude LysMP and crude LysKB317 (Fig. 8).

## Discussion

Amidst the global crisis on antimicrobial resistance and heavy emphasis on responsible use of antibiotics and global collaboration on antimicrobial stewardship programs, recent research has shifted focus towards antibiotic alternatives to combat antibiotic-resistant bacteria [24]. This has drawn attention to the development of bacteriophages and their encoded peptidoglycan hydrolases, also known as endolysins, which specifically target and lyse bacterial cell walls [25] without development of bacterial resistance [15, 17]. Research on endolysins has mostly been focused on human and animal health [26, 27], but the use of endolysins to prevent bacterial contamination with fuel ethanol production has been shown to be an effective strategy [1, 16]. Furthermore, yeast cell surface display of endolysin [39] and direct yeast secretion of endolysin [40] have the potential to improve control of contaminants.

In general, endolysins can be divided into four major classes based on the peptidoglycan cleavage activity [41]: (1) glycosidases cleave glycosidic bonds associated with N-acetylglucosamine (GlcNAc) or N-acetylmuramic acid (MurNAc); (2) amidases cleave the amide bond between the MurNAc and L-alanine residue, the first amino acid of the peptide component of the peptidoglycan (PG); (3) endopeptidases cleave the amide bonds between amino acids present in the PG; and (4) lytic transglycosylases cleave the glycosidic linkages between disaccharide subunits within PG [15]. The group I glycosidases are further divided into two classes: (1) glucosaminidases, which cleave specifically N-acetyl-β-D-glucosamine residues and contiguous monosaccharides in N-glycans, cell walls, and chitin, and (2) muramidases or more commonly known as lysozymes including endolysins such as LysMP, which hydrolyse the β-1,4-glycosidic bonds connecting MurNAc and GlcNAc. Lysozyme activity is attributed by five distinct glycoside hydrolase families, named GH22, GH23, GH24, GH25, GH73 and more recently GH108 was identified [42].

Bioethanol fermentation facilities are inherently prone to bacterial contamination found in raw materials such as corn mash and process water [4, 6, 28, 29] which is difficult to eradicate from facilities [30, 31] even with chemical treatments such as hop acids and chlorine-based oxides [9, 10]. LAB species, in particular, *L. fermentum*, have been found to be one of the primary contaminants that thrive under the conditions typically found in bioethanol fermentation, such as low pH, high glucose concentration, and anaerobic conditions, and can have a negative impact on ethanol-producing yeast, leading to unpredictable acute stuck fermentation [32]. Furthermore, LAB isolated from bioethanol fermentation facilities have demonstrated antibiotic resistance, with some strains harbouring multiple drug-resistant genes against commonly used antibiotics like penicillin G, erythromycin and virginiamycin [31, 33, 35]. This poses a challenge in the treatment of infections in industrial corn ethanol fermentation settings. The economic loss associated with the premature shutdowns of the bioethanol facilities due to stuck fermentation have been well documented [7]. Research and development of bio-mitigation methods for LAB contaminants in fermentation tanks have gradually received more attention, and innovative approaches are being explored to combat such issue. One recent report by Kapetanakis et al. had investigated the possibility of engineering a *S. cerevisiae* strain with knockout mutations of amino acid transporter (Qdr) to limit cross-feeding and propagation of *L. fermentum* [37]. Other research avoided stuck fermentation caused by *Lactiplantibacillus plantarum* using *S. cerevisiae* quorum sensing signal molecule, 2-phenylethanol [38]. However, these methods do not address the source of contamination inside the fermentation tank.

### Biochemical stabilization of LysMP

According to Hadinia et al., LAB such as *L. fermentum* can tolerate up to 6% salinity (or 1 M) solution without negative impact on bacteria [44]. Our result show that LysMP can tolerate NaCl concentration up to 1 M without substantial impact to the lytic activity (Fig. 2A), which is at least equivalent to previously described endolysins [45]. The LysMP showed improved activity in the presence of 300–500 mM NaCl. Previous studies showed that cell wall binding domain of endolysin contains various hydrophobic patches that interact with bacterial peptidoglycan [46]. At higher salt (> 250 mM) concentration, the stable salt bridges may be established between peptidoglycan layer and endolysin and improve catalytic potential of endolysin (Fig. 1B).

Divalent metal cations have been shown to improve lytic activity of endolysins such as *Listeria* targeting endolysin HPL118 and Bacillus targeting endolysin LysB4 [47]. The LysMP activity in the presence of divalent cations were examined based on metal ion-binding site prediction (MIB2; [39]) and protein–ligand binding site prediction** (**COACH; [48]). The structural prediction analyses have revealed the potential presence of a zinc finger domain, with various divalent metal ion binding sites distributed across the endolysin (Additional file 1: Figs. S1, S2). Addition of divalent ions such as calcium, iron and magnesium ions resulted in some improvement of endolysin’s activity (Fig. 4). Nonetheless, the accuracy of the protein–ligand binding prediction model and limited enhancement of activity with copper ion (Fig. 4B) and zinc ion (Fig. 4E) supplementation warrant further evaluation to understand the co-factor requirement for LysMP.

### Lytic potential of LysMP in fermentation

The LysMP endolysin showed efficacy against all tested *L. fermentum* strains and other LAB species such as *L. casei*, *L. rhamnosus*, *L. reuteri* and *L. brevis*, but was ineffective against *L. plantarum*, *L. lactis* and *L. buchneri* (Fig. 5). The LysMP sensitivity profile result was expected and similar to other endolysins previously characterized against bioethanol contaminant isolates [1, 16]. Endolysins have shown the catalytic activity to be either strain or species specific [15, 17, 18, 41]. Traditional corn mash bioethanol fermentation tanks are operated in a temperature range of 30–35 °C with some exception for thermotolerant yeast at 42–45 °C and under the pH range of 4–6 and at ethanol concentration below 25% for up to 48 h or more [5, 51]. Therefore, the LysMP lytic activity should be stable at typical fermentation pH and temperature condition for at least 48 h (Fig. 3B, C). The ethanol tolerance of LysMP remains stable up to 10% for a period of 48 h; however, the enzyme’s stability is significantly reduced at a concentration 20% or greater potentially due to enzyme precipitation (Fig. 3D). Despite LysMP precipitation at high ethanol concentration, it may still be possible to prevent the proliferation of contaminants prior to ethanol concentration reaches 20% (Figs. 2A**,** 6). When exploring contamination mitigation strategies, the complexity of bacterial contamination in bioethanol facilities is not limited to a single strain [6]. Thus, a reasonable intervention strategy should consider employment of endolysin(s) capable of broad-spectrum activity against LAB or combining different endolysins to target problematic LAB. We examined whether the efficacy of using LysMP as an antimicrobial agent in corn mash fermentations could be improved when used in combination with another previously described endolysin, LysKB317 [1]. The results indicated that both enzymes, which were added as crude lysates, were efficacious in controlling *L. fermentum* infection and able to prevent stuck fermentation when compared with no treatment infection control. However, we were not able to demonstrate any synergy or advantage of using the combined enzyme mix (Fig. 8). Phylogenetically, LysMP is closely related to LysKB317 based on protein sequence alignment (Additional file 1: Fig. S3). Structurally, based on predicted protein folding, LysMP and LysKB317 demonstrated differences in the EAD active site, linker length as well as the CBD binding site as observed by the superimposed structure and domains [EAD and CBD; (Additional file 1: Fig. S4)].

### Exogenous addition of LysMP prevents stuck fermentation

Using corn mash fermentations, we confirmed that the addition of LysMP can inhibit *L. fermentum* infection and prevent stuck fermentation (Fig. 5). More than a 3-log reduction in bacterial load was observed in infected corn mash flasks containing LysMP compared to infection control samples without treatment, which had a 2-log increase of bacterial load by the end of the fermentation. The improved glucose utilization and the production of ethanol in the LysMP treated *L. fermentum* challenged fermentations compared to no treatment infection control was substantial and demonstrates the enzyme’s effectiveness with corn mash fermentations. The reduced lactic acid and acetic acid production levels in LysMP treated fermentations (Fig. 7C, D) further support the value of this endolysin for preventing stuck fermentation and restoring the ethanol production in level similar to the uninfected corn mash samples.

## Conclusion

The findings of this study demonstrated the effectiveness of LysMP in preventing stuck fermentation by exogenous addition of the endolysin to treat *L. fermentum* infection in an environment commonly found in fermentation tanks. Bacteriophage-derived endolysin such as LysMP can be a good alternative or supplement to existing antibiotics mitigation strategies to treat LAB contaminations commonly found in fermentation tanks of bioethanol facilities.

## Methods

### Bacterial and yeast strains and culture conditions

*Saccharomyces cerevisiae* NRRL-Y2034 was grown in YPD Broth (BD Biosciences) at 30 °C with aeration at 200 rpm. All LAB strains used in this study (Table 1) were grown in MRS Broth (BD Biosciences) at 30 °C without shaking. *Escherichia coli* strains were grown in LB Broth (BD Biosciences) with 50 µg/mL ampicillin (Amp; Sigma-Aldrich) or 50 µg/mL kanamycin (Kan; Sigma-Aldrich) depending on the plasmid selectable marker.

### Genome annotation and phage lysin-associated domain mining

*L. fermentum* KGL7 genome sequence was assembled and annotated for putative protein coding domain identification using RAST (Rapid Annotation of microbial genomes using Subsystems Technology) [52]. The annotated genome was further searched for domains associated with bacteriophage lysin and peptidoglycan hydrolase found in protein family database (Pfam) such as PF01510: Amidase_2, PF01520: Amidase_3, PF05382: Amidase_5, PF05257: CHAP domain, PF01832: Glucosaminidase, PF01183: Glycoside hydrolase family 25, PF00877: NlpC/P60 family, PF13529: Peptidase_C39 like family, PF01551: Peptidase_M23 family, PF05105: Phage_holin, PF06605: Prophage endopeptidase tail, PF09693: Phage_XkdX, PF08230: CW_7 repeat, PF08924: DUF1906, PF10934: DUF2634 (unknown function associated with phages), and PF03734: L,D-transpeptidase catalytic using KBase [20, 21]. The identified domain homology was further screened for an intact phage-based endolysin gene into the genome and used for the further investigation.

### Constructs and plasmids

*L. fermentum* KGL7 LysMP endolysin gene (LysMP) was codon optimized for *E. coli* expression, synthesized, and cloned into plasmid pET−21a(+) containing 6 × His-Tag at C-terminal by GenScript. Plasmid pET−21a(+) carrying LysMP was transformed into *E. coli* One Shot^™^ BL21(DE3) pLysS Chemically Competent (ThermoFisher Scientific) per manufacturer’s protocol.

### Expression and purification of endolysin

*E. coli* BL21 (DE3) pLysS with pET-21a(+)::LysMP was induced similar to previously discussed methods [1] with 0.5 mM of isopropyl β-D-1-thiogalactopyranoside (IPTG; Sigma-Aldrich) in LB broth and ampicillin (50 µg/mL) overnight at 37 °C with agitation. Cells were harvested by centrifugation at 5000×*g* for 20 min at 4 °C. Cells were then lysed with B-PER (ThermoFisher Scientific) and the addition of 200 µg/mL lysozyme (20 mg/mL in 1 mM Tris–HCl, pH 8.0; ThermoFisher Scientific), DNaseI (10 U/mL; ThermoFisher Scientific), and RNase I (10 U/mL; ThermoFisher Scientific), followed by gentle inversion for 30 min at room temperature. Soluble protein fraction was separated from whole cell lysate via 8000×*g* centrifugation at 4 °C for 20 min and purified using FPLC. The FPLC purification was carried out using an ÄKTA pure^™^ 150 (Cytiva) with a TALON^®^ Superflow^™^ 5 mL cartridge (Cytiva). A 30 mL sample of the clarified cell lysate was loaded at 0.5 mL/min onto the column previously conditioned with 5 column volumes (CV) of equilibration buffer (300 mM NaCl in 50 mM Tris–HCl buffer, pH 7.7) at 5 mL/min. After the lysate loading, the column was washed with 10 CV of equilibration buffer or until the absorbance was below 1.0 mAU at 280 nm. The bound protein was eluted in 5 mL fractions with 10 CV of equilibration buffer supplemented with 200 mM imidazole and 10% glycerol. All collected fraction were analysed on 4% to 15% (w/v) stain free Tris–glycine precast SDS-PAGE [sodium dodecyl sulfate (SDS)-polyacrylamide gel electrophoresis; (Bio-Rad)] as described previously [1]. The buffer was exchanged in the purified protein to remove imidazole using 15 mL Amicon Ultra-15 column with 30 kD molecular cutoff (MilliporeSigma) and 50 mM Tris–HCl buffer (300 mM NaCl and 10% glycerol (v/v), pH 7.0). Buffer was exchanged 3 times leaving the final volume of 1 mL purified LysMP. A polyethersulfone (PES) membrane syringe filter with a 0.22-µm pore size (MilliporeSigma) was used to filter purified enzyme. The purified protein was further quantified using a Qubit 3 fluorometer (ThermoFisher Scientific) and Qubit Protein Assay Kit (ThermoFisher Scientific).

### Spot plate assay

Antimicrobial spot assay of LysMP against *L. fermentum* 0315-25 was performed using polyacrylamide 5% gel. Target bacterial strain was inoculated in 50 mL MRS and grown overnight at 37 °C without shaking, and centrifuge. The culture was centrifuged at 8000×*g* for 10 min and the bacterial pellet was suspended in phosphate buffer (50 mM NaH_2_PO_4_, pH 7.0) and adjusted to OD_600_ = 50. One millilitre of resuspended cells was mixed with 7.50 mL of sterile PBS buffer in a 15 mL Falcon tube. Contents were then mixed with 2 mL of acrylamide/bis (30% (w/v), 200 µL of 10% (w/v), ammonium persulfate (APS), and 50 µL of N,N,N′,N′-tetramethylethylene-diamine (TEMED) and poured into a petri dish (100 × 15 mm) to polymerize. Purified LysMP protein [1 µg/µL; 1 µL] was spotted on the polymerized gel and dried for 10–15 min. Sterile PBS was used as negative control. The plate was then incubated at 37 °C overnight and examined for zone of clearing.

### Growth inhibition assay

Growth inhibition assay was performed at 37 °C for 24 h. Using a SpectraMax M2e Microplate Reader (Molecular Devices) with purified LysMP endolysin. Overnight grown bacteria cultures (Table 1**)** were adjusted to a final OD_600_ = 0.05 in MRS medium. Eighty microliters of cells were mixed with 20 μL of LysMP (treatment; 75 µg/mL to 250 µg/mL) or PBS buffer (pH 7.4; control), and then diluted to 200 µL per well with MRS medium in 96-well microtiter plates (round bottom; Greiner). Plates were incubated at 37 °C without shaking and OD_600_ was measured every 30 min for 24 h using. Treatment and control wells were performed in triplicates to determine growth inhibition compared to control at 24 h.

### Bacterial viability assay

Endolysin activity was confirmed by examining the viability of bacterial cells population using Cellometer X2 image cytometer (Nexcelom) as described by Hodgkin et al. [53]. Ten-microliter of OD_600_ = 0.5 *L. fermentum* 0315-25 (10^7^ CFU/mL) were mixed with 90 μL of 20 mM Tris–Cl buffer (pH 7) containing final concentration of 100 μg/mL LysMP endolysin and incubated for 30 min. Subsequently, 9 μL of sample was mixed with 1 μL of 10 mM SYTOX^®^ and 4 μL was pipetted into a Nexcelom counting chamber (CHT4-SD025), where the inlet and outlet ports were closed with clear tape to prevent evaporation. The bright-field and fluorescent (Excitation: 490 nm/Emission: 530 nm) images were acquired at four different locations in the chamber. The images were analysed by the Cellometer Spectrum software (version 3.2.1.2). Total bacterial concentrations were enumerated based on fluorescent intensities and cell size. Green-stained Limosilactobacilli samples were analysed to enumerate percent of dead cells based on total bacterial concentrations.

### Salt buffer effect on LysMP

Different salt concentrations were assayed to determine the effect on LysMP stability and lytic efficiency in NaCl. The purified LysMP at a final concentration of 100 μg/mL was tested with reaction buffer containing various sodium chloride (NaCl) concentrations (0.1, 0.2, 0.3, 0.4, 0.5, 0.75 M, and 1 M) and *L. fermentum* 0315-25 (10^7^ CFU/mL). Cells were incubated for 30 min at room temperature and lytic activity was examined in a Cellometer as described in bacterial viability assays. Total percent (%) live cells was determined using bacterial viability assay method described above.

### Divalent cation effect on LysMP lytic activity

Based on ligand model prediction, endolysin LysMP may have affinity towards Zn^2+^ [48]. We tested the effect of two different concentrations (0.1 mM and 1 mM) of divalent metal cations (Ca^2+^, Cu^2+^, Fe^2+^, Mg^2+^ and Zn^2+^) on enzyme activity by examining growth inhibition of *L. fermentum* 0315-25 in the presence of LysMP. Target bacteria were grown to OD_600_ = 0.5, washed once with PBS, pH 7.4, and resuspended in 1X PBS. Concentrations of 100 mM of each CaCl_2_, CuCl_2_, FeCl_2_, MgCl_2_, and ZnCl_2_ (Sigma-Aldrich) were separately dissolved in sterile ultrapure water and filtered (0.22 µm; Millipore). A combined volume of 200 µL reaction (20 µL of cell, 20 µL of endolysin at a final concentration of 100 μg/mL or water for negative control, 150 µL MRS, and 10 µL of divalent cation to achieve desired final concentration was added to each well in a clear 96-well (round bottom; Greiner CELLSTAR^®^ microtiter plate. Using a SpectraMax M2e plate reader, OD_600_ was used to measure every 30 min for 9 h at 37 ℃.

### Temperature, pH, and ethanol stability assays

Thermostability of LysMP was determined by incubating 1 mg/mL of purified endolysin for 0.5, 24, and 48 h in 300 mM NaCl, Tris–HCl (Sigma-Aldrich) assay buffer, pH 7 at = 4 °C, 20 °C, 30 °C, 37 °C, 50 °C, 60 °C, and 95 °C before performing the bacterial viability assays at a final enzyme concentration of 100 μg/mL as described above in bacterial viability assay section. Similarly, 1 mg/mL of purified endolysin was used for pH stability at pH 4.0, 5.0, 6.0, 7.0, and 8.0 (21 mM citric acid, 58 mM NaH_2_PO_4_ buffer adjusted to the pH indicated) and ethanol stability in ethanol concentrations of 0–30% (v/v) in reaction buffer, pH 7, for 0.5, 24, and 48 h at 25 °C temperature [1] and examined for bacterial viability activity as described above.

### Preparation of small-scale corn mash fermentation

Corn mash fermentation was performed as described previously with slight modification [1, 5]. *S. cerevisiae* NRRL Y-2034 (Table 1) was grown overnight in YPD broth supplemented with additional glucose (final concentration 7% w/v) at 30 °C with 200 rpm shaking. The contaminant *L. fermentum* strain 0315-25 (Table 1) was grown in MRS media with 5% glucose (w/v) at 30 °C without shaking to mid-log phase (OD_600_ nm = 0.6–0.8). Both yeast and bacteria cells were collected via centrifugation and resuspended in sterile phosphate buffered saline (PBS; pH 7.4, Fisher Scientific) to OD_600_ = 1 for yeast, and OD_600_ = 4 for *L. fermentum* 0315-25, respectively. One OD_600_ is approximately 6 × 10^7^ CFU/mL for yeast and 1 × 10^8^ CFU/mL for bacteria. Corn mash (approximately 33% solids) was collected from a commercial dry-grind ethanol facility and stored at − 20 °C until use and autoclaved before use. In separate 25-mL Erlenmeyer flasks, 16 mL corn mash with ammonium sulfate (0.12%, w/v; Sigma-Aldrich) and glucoamylase (10 μL of Alcoholase II Liquid 30098-LS341-Glucoamylase) was dispensed and incubated overnight for liquefaction at 40 °C and 100 rpm shaking overnight. Three sets of flasks in duplicate were prepared and designated as yeast control, bacterial contamination challenge and bacterial contamination challenge with treatment. All the flasks were inoculated with 0.1 mL *S. cerevisiae* inoculum. While 0.4 mL challenged bacterial inoculum were added into the flasks except yeast control designated flasks. The purified endolysin LysMP was added into the treatment flasks at a final concentration of 250 µg/mL. In each flask, 1 mL MRS broth supplemented with 5% glucose (w/v) was added to promote growth of challenge bacteria. Sterile water was added to the final volume of 20 mL. The flasks were plugged with a rubber stopper containing a 20-gauge 0.9 mm × 40 mm Precision Glide needle (Becton Dickinson) to vent excess CO_2_ and incubated at 30 °C with 100 rpm shaking for 48 h. Samples (0.5 mL) were taken at 0, 24, and 48 h and diluted in PBS (pH 7.4). The bacterial count for each pooled sample was performed on 1.5% MRS agar plates and yeast inhibitor (10 µg/mL; cycloheximide) by spiral serial dilution using the Eddy Jet 2 spiral plater (IUL Instruments) set in the E mode 50 (50 µL sample). Plates were incubated anaerobically using the Anaero Pack System (Mitsubishi) at 37 °C for 18 h [16]. Colony forming unit/mL (CFU/mL) were enumerated using a Flash & Go plate reader (IUL Instruments) with minimum of detection for sample at > 3 − log_10_ (CFU/mL). As previously described, a high-performance liquid chromatography (HPLC) system with 300 mm Aminex HPX 87H column (Bio-Rad laboratories, Inc.) was used to quantify presence of acetic acid, glucose, lactic acid and ethanol [54].

### Testing synergistic effects of endolysin treatment

Overnight expression of LysMP and LysKB317 were induced and cell harvested in conditions mentioned above except for LysKB317 expressing cell (E. cloni 10 G/pRham N-His Kan::LysKB317; Table 1), 0.2% (w/v) L-rhamnose (Sigma-Aldrich) was used for induction instead of 0.5 mM IPTG [1]. Crude endolysin LysMP and LysKB317 were extracted by sonication (135 s/50 amplitude/15 s pulse followed by 30 s rest; Fisherbrand^™^ Model 705). Concentration of crude endolysins were estimated by densitometry using a ChemiDoc imager on SDS-PAGE gel bands. Crude lysate has an estimated (250 µg/mL) endolysin(s) (LysMP and/or LysKB317) were added into 20 mL corn mash fermentation.

### Statistical analysis

Two-way analysis of variance (ANOVA) with Tukey post hoc was applied to where appropriate to analyse the experimental results where sample are performed in at least two independent biological replicates (*n* = 2). Statistical significance is determined by *p < 0.05, **p < 0.01, ****p < 0.0001 (GraphPad Prism version 9.5.1).

### Supplementary Information

**Additional file 1: Figure S1.** Metal ion binding prediction of endolysin LysMP. The prediction tool MIB2 was used to predict possible metal ion bindings [1]. A Predicted Zn^2+^ binding sites on LysMP. B Predicted Zn^2+^ metal ion binding potential based on the amino acid sequence of LysMP. **Figure S2.** Protein-ligand binding prediction of LysMP. Protein-ligand binding site (COACH) prediction of LysMP. Predicted binding site amino-acid residues at 33, 100, 102, 128, 152, and 154 [2]. **Figure S3.** Phylogenetic tree of endolysins. Multiple sequence alignment of endolysins using Clustal Omega and phylogenetic tree generated using the tree viewer [3]. **Figure S4.** Superimposed predicted endolysin structures. Protein prediction structure of LysMP (Red) and LysKB317 (Blue) and for enzymatically active domain (EAD) and cell wall binding domain (CBD) using UCSF ChimeraX [4]. Predicted endolysin structures were generated using ESMFold [5].

## Data Availability

Data and materials will be available upon reasonable request.

## Abbreviations

ANOVA: Analysis of variance
CFU: Colony forming unit
CV: Column volume
CBD: Cell wall binding domain
EAD: Enzymatically active domain
FPLC: Fast protein liquid chromatography
HPLC: High-performance liquid chromatography
IPTG: Isopropyl-β-D-1-thiogalactopyranoside
LAB: Lactic acid bacteria
LB: Luria–Bertani
MRS: De Man, Rogosa and Sharp
OD: Optical density
PBS: Phosphate-buffered saline
RPM: Revolution per minute
SDS-PAGE: Sodium dodecyl sulfate-polyacrylamide gel electrophoresis
TEMED: N,N,N′,N′-Tetramethyl ethylenediamine
YPD: Yeast extract peptone dextrose
