# Supplementary material for: Novel endolysin LysMP for control of Limosilactobacillus fermentum contamination in small-scale corn mash fermentation
Source: Biotechnol Biofuels Bioprod. 2023 Sep 29;16:144. doi: 10.1186/s13068-023-02400-5 (PMC10541714; doi:10.1186/s13068-023-02400-5)
Supplement: Supplementary file 1 — Additional file 1: Figure S1. Metal ion binding prediction of endolysin LysMP. The prediction tool MIB2 was used to predict possible metal ion bindings [1]. A Predicted Zn2+ binding sites on LysMP. B Predicted Zn2+ metal ion binding potential based on the amino acid sequence of LysMP. Figure S2. Protein-ligand binding prediction of LysMP. Protein-ligand binding site (COACH) prediction of LysMP. Predicted binding site amino-acid residues at 33, 100, 102, 128, 152, and 154 [2]. Figure S3. Phylogenetic tree of endolysins. Multiple sequence alignment of endolysins using Clustal Omega and phylogenetic tree generated using the tree viewer [3]. Figure S4. Superimposed predicted endolysin structures. Protein prediction structure of LysMP (Red) and LysKB317 (Blue) and for enzymatically active domain (EAD) and cell wall binding domain (CBD) using UCSF ChimeraX [4]. Predicted endolysin structures were generated using ESMFold [5]. [file 13068_2023_2400_MOESM1_ESM.docx]

**Additional Materials**

**Novel Endolysin LysMP for Control of *Limosilactobacillus* *fermentum* Contamination in Small-Scale Corn Mash Fermentation**

Maulik H. Patel^1^, Shao Y. Lu^2*^, Siqing Liu^2^, and Christopher D. Skory^2^.

^1^Oak Ridge Institute for Science and Education (ORISE), USA

^2^USDA, Agricultural Research Service, National Center for Agricultural Utilization Research, Renewable Product Technology Research Unit, 1815 N. University St., Peoria, IL, 61604 USA

*Corresponding author: Shao-Yeh Lu, Ph.D., USDA-ARS-NCAUR, 1815 N. University St., Peoria, IL 61604-3902, USA; (309) 681-6067; shao.lu@usda.gov

**Figure S1. Metal ion binding prediction of endolysin LysMP.** The prediction tool MIB2 was used to predict possible metal ion bindings [1]. **A.** Predicted Zn^2+^ binding sites on LysMP. **B.** Predicted Zn^2+^ metal ion binding potential based on the amino acid sequence of LysMP.


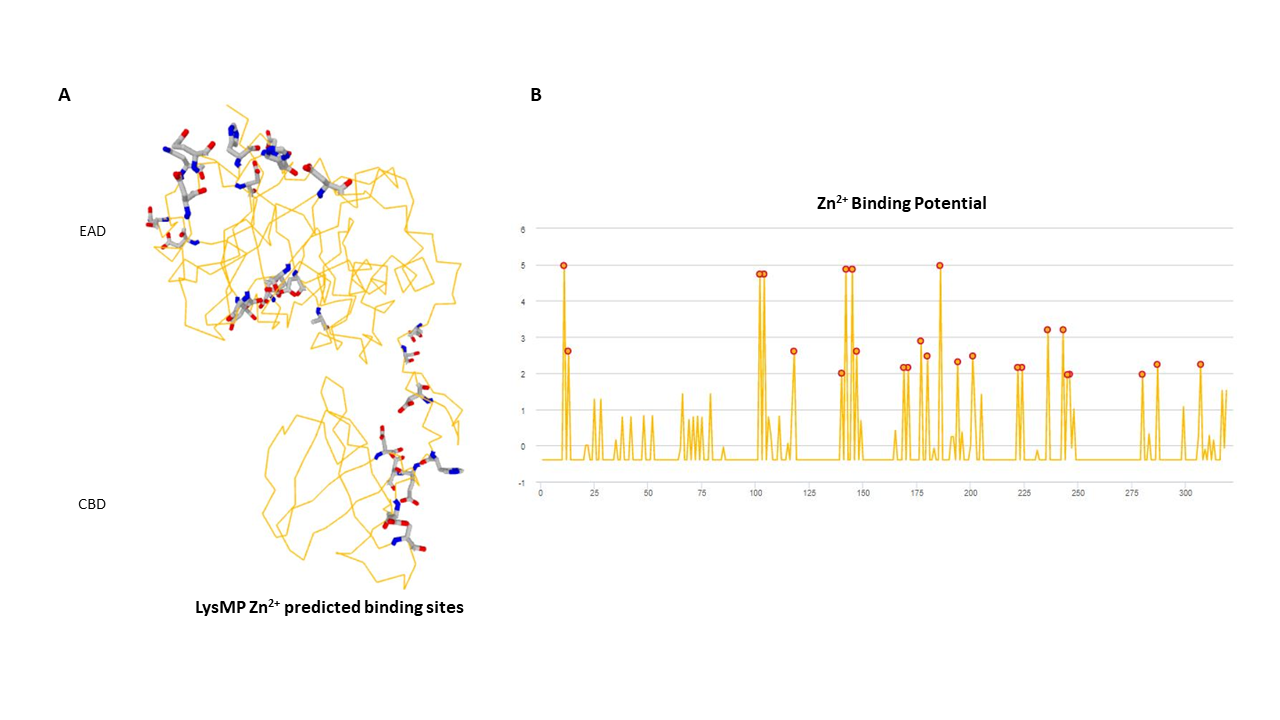


**Figure S2. Protein-ligand binding prediction of LysMP.** Protein-ligand binding site (COACH) prediction of LysMP. Predicted binding site amino-acid residues at 33, 100, 102, 128, 152, and 154 [2].


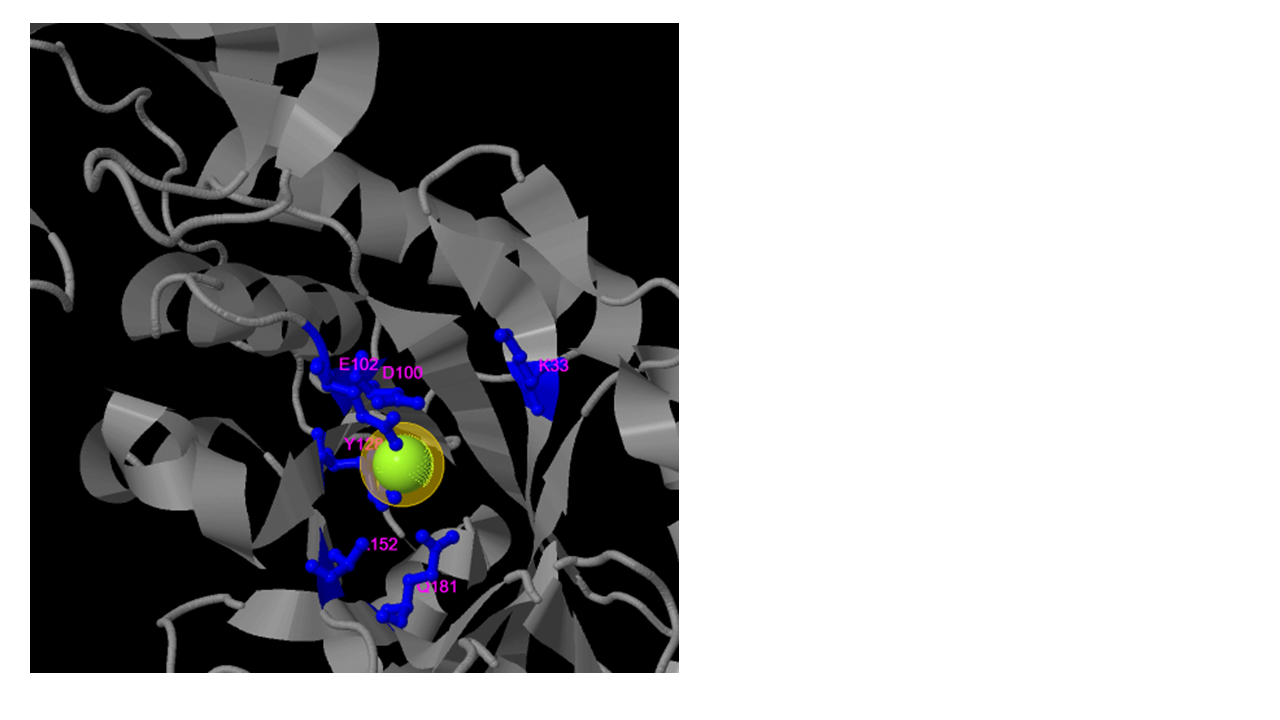


**Figure S3. Phylogenetic tree of endolysins.** Multiple sequence alignment of endolysins using Clustal Omega and phylogenetic tree generated using the tree viewer [3].


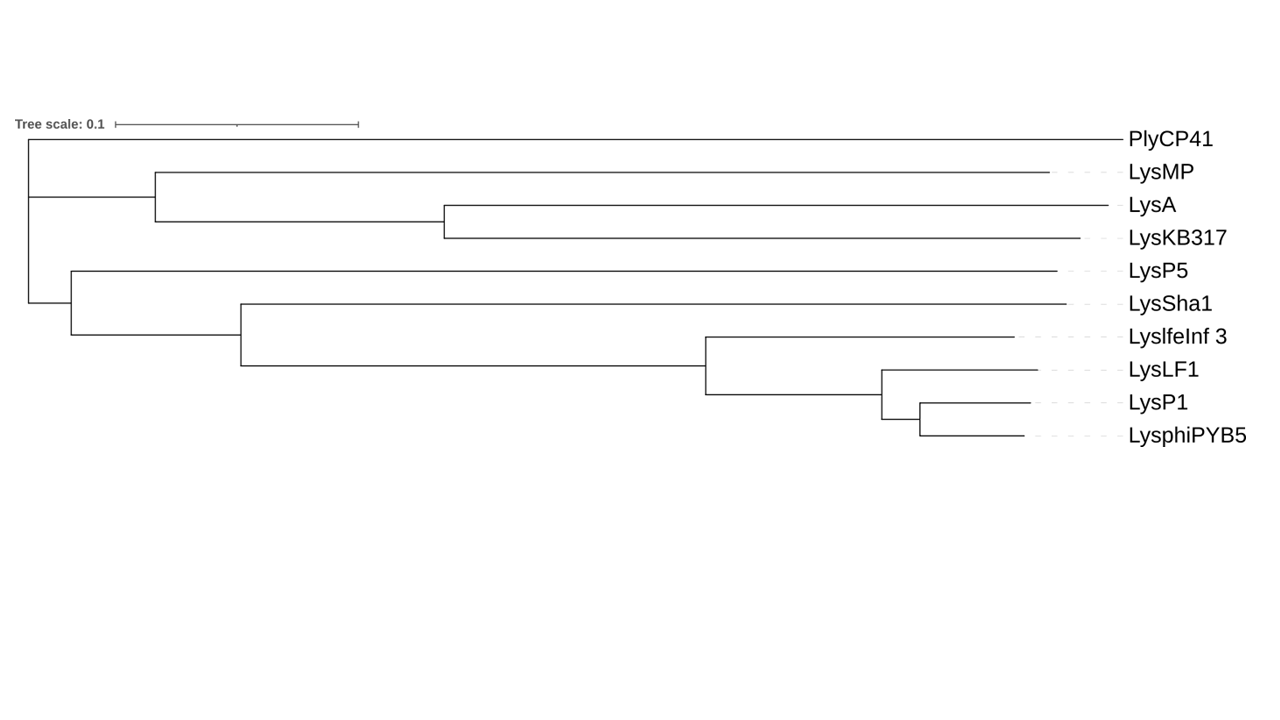


**Figure S4. Superimposed predicted endolysin structures.** Protein prediction structure of LysMP (Red) and LysKB317 (Blue) and for enzymatically active domain (EAD) and cell wall binding domain (CBD) using UCSF ChimeraX [4]. Predicted endolysin structures were generated using ESMFold [5].

**
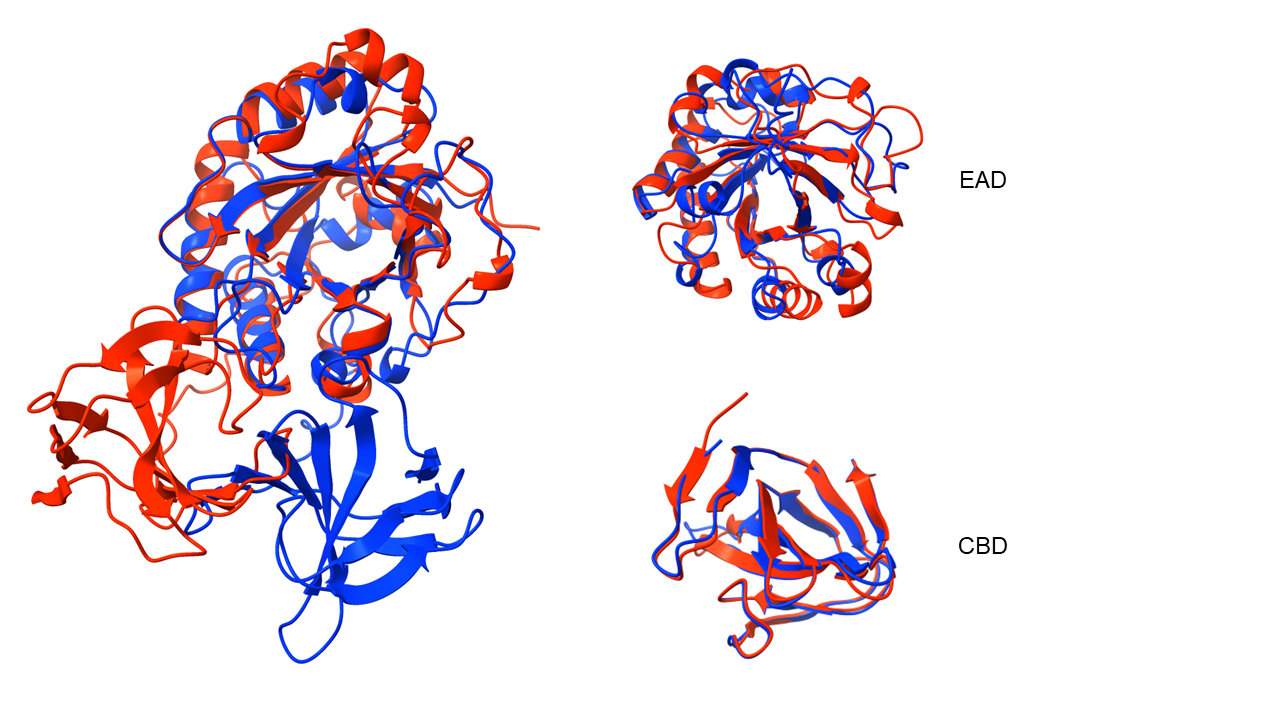
**

**REFERENCE**

[1] Yang J, Roy A, Zhang Y: Protein–ligand binding site recognition using complementary binding-specific substructure comparison and sequence profile alignment. Bioinformatics 2013, 29(20):2588-2595.

[2] Zhang C, Freddolino PL, Zhang Y: COFACTOR: improved protein function prediction by combining structure, sequence and protein–protein interaction information. Nucleic acids research 2017, 45(W1):W291-W299.

[3] Madeira F, Pearce M, Tivey ARN, Basutkar P, Lee J, Edbali O, Madhusoodanan N, Kolesnikov A, Lopez R: Search and sequence analysis tools services from EMBL-EBI in 2022. Nucleic Acids Research 2022, 50(W1):W276-W279.

[4] Pettersen EF, Goddard TD, Huang CC, Meng EC, Couch GS, Croll TI, Morris JH, Ferrin TE: UCSF ChimeraX: Structure visualization for researchers, educators, and developers. Protein Science 2021, 30(1):70-82.

[5] Lin Z, Akin H, Rao R, Hie B, Zhu Z, Lu W, Smetanin N, Verkuil R, Kabeli O, Shmueli Y: Evolutionary-scale prediction of atomic-level protein structure with a language model. Science 2023, 379(6637):1123-1130.
